# Supplementary material for: Evaluation of the effect of RNA secondary structure on Cas13d-mediated target RNA cleavage
Source: Mol Ther Nucleic Acids. 2024 Jul 20;35(3):102278. doi: 10.1016/j.omtn.2024.102278 (PMC11364014; doi:10.1016/j.omtn.2024.102278)
Supplement: Document S1. Tables S1 and S2 [file mmc1.pdf]

OMTN, Volume *35*

Supplemental information

Evaluation of the effect

of RNA secondary structure

on Cas13d-mediated target RNA cleavage

Mouraya Hussein, Ye Liu, Monique Vink, Pascal Z. Kroon, Atze T. Das, Ben Berkhout, and Elena Herrera-Carrillo

Table S1. DNA oligonucleotides

Oligonucleotide name	Oligonucleotide sequence
FWD 5'UTR-Leader-crRNA	AAACGAGAACAGATCTACAAGAGATCG
REV 5'UTR-Leader-crRNA	AAAACGATCTCTTGTAGATCTGTTCTC
FWD RdRp-crRNA	AAACGCTATGTAAGTTTACATCCTGAT
REV RdRp-crRNA	AAAAATCAGGATGTAAACTTACATAGC
FWD-NC-crRNA	AAACGAGGAGGTACTGCTGCGATGGGA
REV-NC-crRNA	AAAATCCCATCGCAGCAGTACCTCCTC
FWD 5'UTR-Leader-shRNA	AGCTTTTCCAAAAAACGATCTCTTGTAGATCTGTTCTCTCTTGAAGAACAGATCTACAAGAGATCGGGG
REV 5'UTR-Leader-shRNA	GATCCCCCGATCTCTTGTAGATCTGTTCTTCAAGAGAGAACAGATCTACAAGAGATCGTTTTTTGGAAA
FWD RdRp-shRNA	AGCTTTTCCAAAAAAATCAGGATGTAAACTTACATATCTCTTGAATATGTAAGTTTACATCCTGATGGG
REV RdRp-shRNA	GATCCCCATCAGGATGTAAACTTACATATTCAAGAGATATGTAAGTTTACATCCTGATTTTTTTGGAAA
FWD-NC-shRNA	AGCTTTTCCAAAAAATCCCATCGCAGCAGTACCTCCTCTCTTGAAGGAGGTACTGCTGCGATGGGAGGG
REV-NC-shRNA	GATCCCCTCCCATCGCAGCAGTACCTCCTTCAAGAGAGGAGGTACTGCTGCGATGGGATTTTTTGGAAA

Table S2. Target oligonucleotide sequences

Oligonucleotide name	Oligonucleotide sequence
RdRp.WT.FWD	AATTCAGGTGTTGTACATAATCAGGATGTAAACTTACATAGCTCTAGACTTAGTTTCTGCA
RdRp.WT.Rev	GAAACTAAGTCTAGAGCTATGTAAGTTTACATCCTGATTATGTACAACACCTG
RdRp.MUT1R.FWD	AATTCGCTATGTAAGTTTACATCCTGATaagaaATCAGGATGTAAACTTACATAGCCTGCA
RdRp.MUT1R.Rev	GGCTATGTAAGTTTACATCCTGATttcttATCAGGATGTAAACTTACATAGCG
RdRp.MUT2R.FWD	AATTCGTTATGTAAGTTTATATTTTGATaagaaATCAGGATGTAAACTTACATAGCCTGCA
RdRp.MUT2R.Rev	GGCTATGTAAGTTTACATCCTGATttcttATCAAAATATAAACTTACATAACG
RdRp.Mut3R.FWD	AATTCGCTATGTAAATTTAAATCATGATaagaaATCAGGATGTAAACTTACATAGCCTGCA
RdRp.Mut3R.Rev	GGCTATGTAAGTTTACATCCTGATttcttATCATGATTTAAATTTACATAGCG
RdRp.Mut4R.FWD	AATTCGCTCAGTAAATTTAAATCATGATaagaaATCAGGATGTAAACTTACATAGCCTGCA
RdRp.Mut4R.Rev	GGCTATGTAAGTTTACATCCTGATttcttATCATGATTTAAATTTACTGAGCG
RdRp.Mut5R.FWD	AATTCGCAATGCAAATCTAAATCATGAAaagaaATCAGGATGTAAACTTACATAGCCTGCA
RdRp.Mut5R.Rev	GGCTATGTAAGTTTACATCCTGATttcttTTCATGATTTAGATTTGCATTGCG
RdRp.Mut1L.FWD	AATTCATCAGGATGTAAACTTACATAGCaagaaGCTATGTAAGTTTACATCCTGATCTGCA
RdRp.Mut1L.Rev	GATCAGGATGTAAACTTACATAGCttcttGCTATGTAAGTTTACATCCTGATG
RdRp.Mut2L.FWD	AATTCATCAGGATGTAAACTTACATAGCaagaaGTTATGTAAGTTTATATTTTGATCTGCA
RdRp.Mut2L.Rev	GATCAAAATATAAACTTACATAACttcttGCTATGTAAGTTTACATCCTGATG
RdRp.Mut3L.FWD	AATTCATCAGGATGTAAACTTACATAGCaagaaGCTATGTAAATTTAAATCATGATCTGCA
RdRp.Mut3L.Rev	GATCATGATTTAAATTTACATAGCttcttGCTATGTAAGTTTACATCCTGATG
RdRp.Mut4L.FWD	AATTCATCAGGATGTAAACTTACATAGCaagaaGCTCAGTAAATTTAAATCATGATCTGCA
RdRp.Mut4L.Rev	GATCATGATTTAAATTTACTGAGCttcttGCTATGTAAGTTTACATCCTGATG
RdRp.Mut5L.FWD	AATTCATCAGGATGTAAACTTACATAGCaagaaGCAATGCAAATCTAAATCATGAACTGCA
RdRp.Mut5L.Rev	GTTCATGATTTAGATTTGCATTGCttcttGCTATGTAAGTTTACATCCTGATG
5'UTR-Leader.WT.FWD	AATTCAACCAACCAACTTTCGATCTCTTGTAGATCTGTTCTCTAAACGAACTTTAACTGCA
5'UTR-Leader.WT.Rev	GTTAAAGTTCGTTTAGAGAACAGATCTACAAGAGATCGAAAGTTGGTTGGTTG
5'UTR-Leader.Mut1R.FWD	AATTCGAGAACAGATCTACAAGAGATCGaagaaCGATCTCTTGTAGATCTGTTCTCCTGCA
5'UTR-Leader.Mut1R.Rev	GGAGAACAGATCTACAAGAGATCGttcttCGATCTCTTGTAGATCTGTTCTCG
5'UTR-Leader.Mut2R.FWD	AATTCGAGAAtAGATtTAtAAGAGATtGcaccaCGATCTCTTGTAGATCTGTTCTCCTGCA
5'UTR-Leader.Mut2R.Rev	GGAGAACAGATCTACAAGAGATCGtggtgCaATCTCTTaTAaATCTaTTCTCG
5'UTR-Leader.Mut3R.FWD	AATTCGAGAAaAGATaTAgAAGAGATaGcaccaCGATCTCTTGTAGATCTGTTCTCCTGCA
5'UTR-Leader.Mut3R.Rev	GGAGAACAGATCTACAAGAGATCGtggtgCtATCTCTTcTAtATCTtTTCTCG
5'UTR-Leader.Mut4R.FWD	AATTCGAGAtaAGATaTAgAAGAaATaGcaccaCGATCTCTTGTAGATCTGTTCTCCTGCA
5'UTR-Leader.Mut4R.Rev	GGAGAACAGATCTACAAGAGATCGtggtgCtATtTCTTcTAtATCTtaTCTCG
5'UTR-Leader.Mut5R.FWD	AATTCGAtAtaAGATaTAgAAGAaATaGcaccaCGATCTCTTGTAGATCTGTTCTCCTGCA
5'UTR-Leader.Mut5R.Rev	GGAGAACAGATCTACAAGAGATCGtggtgCtATtTCTTcTAtATCTtaTaTCG
5'UTR-Leader.Mut1L.FWD	AATTCCGATCTCTTGTAGATCTGTTCTCaagaaGAGAACAGATCTACAAGAGATCGCTGCA
5'UTR-Leader.Mut1L.Rev	GCGATCTCTTGTAGATCTGTTCTCttcttGAGAACAGATCTACAAGAGATCGG
5'UTR-Leader.Mut2L.FWD	AATTCCGATCTCTTGTAGATCTGTTCTCaagaaGAGAAtAGATtTAtAAGAGATtGCTGCA
5'UTR-Leader.Mut2L.Rev	GCaATCTCTTaTAaATCTaTTCTCttcttGAGAACAGATCTACAAGAGATCGG
5'UTR-Leader.Mut3L.FWD	AATTCCGATCTCTTGTAGATCTGTTCTCaagaaGAGAAaAGATaTAgAAGAGATaGCTGCA
5'UTR-Leader.Mut3L.Rev	GCtATCTCTTcTAtATCTtTTCTCttcttGAGAACAGATCTACAAGAGATCGG
5'UTR-Leader.Mut4L.FWD	AATTCCGATCTCTTGTAGATCTGTTCTCaagaaGAGAtaAGATaTAgAAGAaATaGCTGCA
5'UTR-Leader.Mut4L.Rev	GCtATtTCTTcTAtATCTtaTCTCttcttGAGAACAGATCTACAAGAGATCGG
5'UTR-Leader.Mut5L.FWD	AATTCCGATCTCTTGTAGATCTGTTCTCcaccaGAtAtaAGATaTAgAAGAaATaGCTGCA
5'UTR-Leader.Mut5L.Rev	GCtATtTCTTcTAtATCTtaTaTCtggtgGAGAACAGATCTACAAGAGATCGG
