# Supplementary material for: Evaluation of the effect of RNA secondary structure on Cas13d-mediated target RNA cleavage
Source: Mol Ther Nucleic Acids. 2024 Jul 20;35(3):102278. doi: 10.1016/j.omtn.2024.102278 (PMC11364014; doi:10.1016/j.omtn.2024.102278)
Supplement: Document S2. Article plus supplemental information [file mmc2.pdf]

Molecular Therapy
Nucleic Acids
Original Article

Evaluation of the effect of RNA secondary structure on Cas13d-mediated target RNA cleavage

Mouraya Hussein,[1,2,3] Ye Liu,[1,2,3] Monique Vink,[1,2] Pascal Z. Kroon,[1,2] Atze T. Das,[1,2] Ben Berkhout,[1,2] and Elena Herrera-Carrillo[1,2]

[1]Amsterdam UMC, University of Amsterdam, Medical Microbiology and Infection Prevention, Meibergdreef 9, Amsterdam, the Netherlands; [2]Amsterdam Institute for Immunology and Infectious Diseases, Amsterdam, the Netherlands

The clustered regularly interspaced short palindromic repeats (CRISPR)-Cas13d system was adapted as a powerful tool for targeting viral RNA sequences, making it a promising approach for antiviral strategies. Understanding the influence of template RNA structure on Cas13d binding and cleavage efficiency is crucial for optimizing its therapeutic potential. In this study, we investigated the effect of local RNA secondary structure on Cas13d activity. To do so, we varied the stability of a hairpin structure containing the severe acute respiratory syndrome coronavirus 2 (SARS-CoV-2) target sequence, allowing us to determine the threshold RNA stability at which Cas13d activity is affected. Our results demonstrate that Cas13d possesses the ability to effectively bind and cleave highly stable RNA structures. Notably, we only observed a decrease in Cas13d activity in the case of exceptionally stable RNA hairpins with completely base-paired stems, which are rarely encountered in natural RNA molecules. A comparison of Cas13d and RNA interference (RNAi)-mediated cleavage of the same RNA targets demonstrated that RNAi is more sensitive for local target RNA structures than Cas13d. These results underscore the suitability of the CRISPR-Cas13d system for targeting viruses with highly structured RNA genomes.

INTRODUCTION

Prokaryotes have developed clustered regularly interspaced short palindromic repeats (CRISPR) along with CRISPR-associated (Cas) proteins to defend their genome against invading genetic elements.[1–3] Since the discovery of the CRISPR-Cas system in bacteria and archaea, multiple Cas endonucleases have been identified and characterized, including Cas9, Cas12, and Cas13.[4–7] Unlike the DNA-targeting endonucleases Cas9 and Cas12, the Cas13 enzyme is an RNA-targeting CRISPR effector.[8,9] Cas13 is composed of a recognition (REC) lobe and a nuclease (NUC) lobe. The NUC lobe contains two conserved higher eukaryote and prokaryote nucleotide-binding (HEPN) domains (HEPN1 and HEPN2) that dictate CRISPR RNA (crRNA) maturation and target cleavage.[10–12] The REC lobe is also responsible for processing the precursor crRNA transcript into mature crRNA, which contains the spacer region specific to the target RNA.[13] Specific binding of the Cas13:crRNA complex to the complementary target RNA leads to the formation of a ternary complex that undergoes conformational changes, enabling nuclease activation.[10,13,14] As a consequence, the targeted RNA is cleaved (*cis*-cleavage). *Trans*-cleavage of non-specific bystander RNAs, also known as collateral activity, has also been reported but only *in vitro* and in bacteria.[15–17] Cas13 collateral activity in mammalian cells is still debated, as it was not reported in multiple *in vivo* studies[18–20] but was suggested by another study.[21]

The Cas13 protein family of RNases consists of four well-characterized variants, Cas13a (formerly known as c2c2), Cas13b, Cas13c, and Cas13d, as well as two newly identified variants, Cas13X and Cas13Y.[8,15,22–24] Cas13a, Cas13b, and Cas13d exhibit efficient RNA cleavage activity in mammalian cells. The potential to use members of the CRISPR-Cas13 family for targeting the RNA genome of pathogenic viruses has garnered considerable interest, particularly in the context of the COVID-19 pandemic.[25,26] Our laboratory and others reported promising results regarding the potential of CRISPR-Cas13 as an antiviral approach for severe acute respiratory syndrome coronavirus 2 (SARS-CoV-2).[27–29] Despite the rapid development and regulatory approval of multiple effective vaccines, including viral-vector-based and RNA-based vaccines, challenges persist due to the emergence of SARS-CoV-2 variants of concern (VOCs) and the potential for new (corona) viral outbreaks in the future.[30,31] Recent data indicate a decrease in the neutralization potency of antibodies from recipients of SARS-CoV-2 vaccines against these VOCs, particularly the recent Omicron variant.[32] The US Food and Drug Administration and European Medicines Agency have approved multiple antiviral drugs (remdesivir, tocilizumab, baricitinib, and Paxlovid) for COVID-19, but there remains a need for antiviral

Received 1 September 2023; accepted 16 July 2024;
https://doi.org/10.1016/j.omtn.2024.102278.

[3]These authors contributed equally

Correspondence: Elena Herrera-Carrillo, Amsterdam UMC, University of Amsterdam, Medical Microbiology and Infection Prevention, Meibergdreef 9, Amsterdam, the Netherlands.
E-mail: e.herreracarrillo@amsterdamumc.nl

© 2024 The Author(s). Published by Elsevier Inc. on behalf of The American Society of Gene and Cell Therapy. This is an open access article under the CC BY license (http://creativecommons.org/licenses/by/4.0/).

strategies that are easily adaptable to counter rapidly evolving viruses. Therefore, alternative approaches to combat SARS-CoV-2, such as the use of CRISPR-Cas13-based approaches, merit our attention.

Some Cas13 proteins, such as RfxCas13d, possess the ability to target any sequence of interest because they depend solely on crRNA complementarity without the requirement for a protospacer flanking sequence adjacent to the target sequence. This flexibility allows for targeting a wide range of sequences from diverse viral pathogens.[22,33,34] This feature also enables the targeting of highly conserved sequences that are present in most viral isolates, thereby increasing the breadth of the antiviral therapy. Such highly conserved sequences generally encode important viral protein domains that are usually crucial for viral replication.[35,36] These conserved regions are less likely to tolerate escape mutations, implying that viral escape is restricted when such conserved regions are targeted.

The local secondary structure of the targeted RNA molecule is potentially an important factor to consider when designing RNA-based antiviral strategies, as previously demonstrated for RNA interference (RNAi) and antisense oligonucleotides.[37–42] Cas13-crRNAs anneal to the complementary target RNA, and the local RNA structure may affect the action of Cas13 by making the target region less accessible for the crRNA. Several studies indeed indicated an inhibitory effect of target RNA structure on LshCas13a activity.[8,43] However, the impact of RNA structure on Cas13 activity has not been systematically evaluated.

The potential suppressive effect of local RNA structure on Cas13 activity may be particularly important when targeting the structured RNA genome of SARS-CoV-2. For instance, this viral RNA exhibits nearly twice the propensity to form stable RNA structures than the structured RNA genome of the hepatitis C virus.[44] Genome-wide RNA structure analysis of the SARS-CoV-2 genome revealed a multitude of structured RNA motifs, including the ribosomal frameshift element and motifs in the 5′ and 3′ untranslated regions (UTRs).[45–47] Many of these elements exhibit high sequence conservation among virus isolates, likely because they have important roles in viral replication[46] and are therefore potential targets in antiviral strategies.[48,49]

In order to develop an effective CRISPR-Cas13d-based therapy for targeting SARS-CoV-2, a detailed investigation of the influence of RNA structure on Cas13d activity is important. We therefore determined the threshold RNA stability at which Cas13d targeting is impeded in cell culture and *in vitro* experiments. Furthermore, we compared the sensitivity of Cas13d and RNAi cleavage for local target RNA structure.

RESULTS

Systematic destabilization of RNA structure in Cas13d target sequences

We systematically investigated the effect of target RNA structure on CRISPR-Cas13d efficiency. Two potent anti-SARS-CoV-2 crRNA molecules were selected from a previous study and their target regions cloned into the luciferase reporter (Figure 1A).[28] One crRNA targets a sequence within the 5′ UTR-leader sequence, and the other targets sequences that encode the RNA-dependent RNA polymerase (RdRp). The predicted RNA secondary structure of these two targets, with 14-nt flanking the target on both sides, is depicted as the wild-type (WT) structure in Figures 1B and 1C. The thermodynamic RNA stability ΔG (in kcal/mol) of these structures is indicated. The 23-nt regions that are complementary to the designed antiviral crRNAs are highlighted in gray. We designed progressive mutations in the flanks to fold these targets in a stable hairpin structure with a 23-bp stem and a 5-nt loop (AAGAA). This will allow us to measure a possible negative effect of the local template RNA structure on Cas13 action. As we also wanted to investigate the effect of the actual position of the 23-nt target in such a hairpin structure, we generated two maximally stabilized mutants for both targets in which the target sequence is placed on either the 5′ or 3′ side of the base-paired stem segment, which were designated 1L and 1R, respectively. The thermodynamic stability (ΔG) values of the predicted RdRp 1L and 1R hairpins are −35.9 and −35.4 kcal/mol, respectively (Figure 1B). The ΔG values of the predicted 1L and 1R hairpins of the 5′ UTR-leader target are slightly lower, −39.4 and −39.0 kcal/mol, respectively. We subsequently destabilized these perfect hairpins by mutation of the flanking sequences (mutations encircled in mutants 2R–5R and 2L–5L, Figures 1B and 1C), thus maintaining perfect complementarity of the 23-nt target sequence to the designed crRNAs. For each target, we designed two sets of 10 mutants in which the target RNA hairpin was gradually destabilized. In the 1L and 2R mutants, the hairpins were destabilized by replacing G-C base pairs with G-U wobble pairs. In the 3R–5R and 3L–5L mutants, nucleotide substitutions were introduced that open specific base pairs in the double-stranded stem. Overall, the ΔG values of the hairpin structure gradually increased to a value close to that of the WT structures (ΔG −3.6 and −5.8 kcal/mol for RdRp and 5′ UTR-leader, respectively).

Low sensitivity of Cas13d for secondary structure at the RNA target site

To assess the impact of target RNA structure on the efficiency of Cas13d binding and cleavage (which we define as targeting efficiency) in transfected cells, we employed a luciferase reporter system. In this system, the SARS-CoV-2 target sequences (Figures 1B and 1C) were cloned downstream of the luciferase gene (Figure 1A) such that Cas13d-mediated RNA cleavage will result in a loss of luciferase production. To quantify the CRISPR-Cas13d targeting of the structurally distinct targets, we co-transfected the luciferase reporter constructs with a Cas13d- and crRNA-expressing construct into human embryonic kidney (HEK)293T cells and measured the luciferase level at 48 h post-transfection. As a negative control (NC), we used a non-targeting crRNA that does not target the SARS-CoV-2 target construct or any cellular sequence. For every target variant, the luciferase activity measured with this NC was set at 100% and the relative luciferase level obtained with the targeting crRNA was calculated (Figures 2A and 2C). This relative luciferase expression level was then plotted

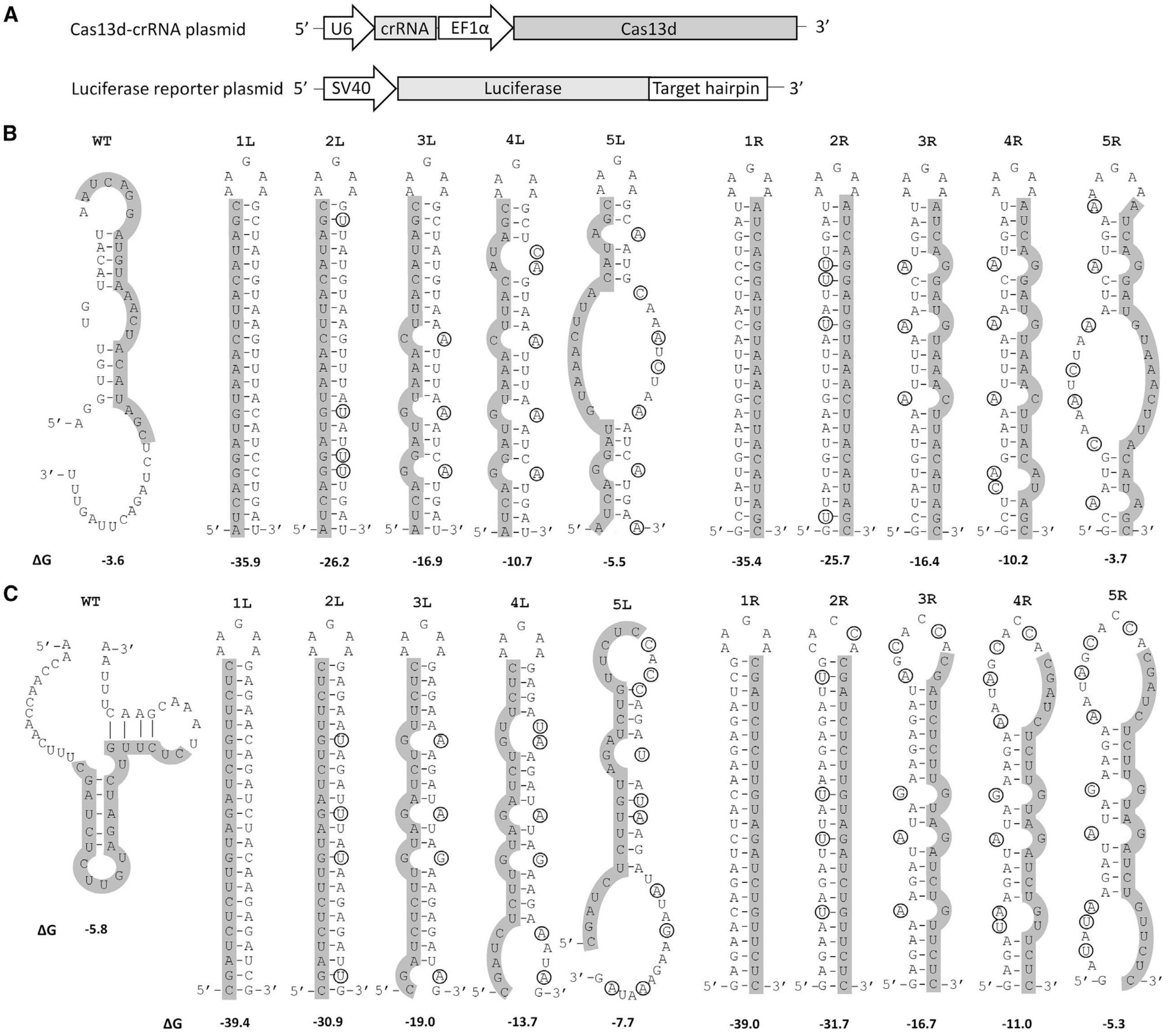

Figure 1. Design of target RNA structures

(A) Schematic representation of the expression plasmid used for Cas13d and crRNA expression (top) and the firefly luciferase reporter (bottom). The RNA polymerase III (RNA Pol III) human U6 promoter drives crRNA transcription, while Cas13d expression is driven by the RNA Pol II EF1α core promoter. The SARS-CoV-2 target sequences were inserted downstream of the firefly luciferase gene under the control of the SV40 promoter in the pGL3 reporter plasmid. (B and C) Predicted RNA structures of the wild-type (WT) RdRp (B) and 5′ UTR-leader (C) target sequences, along with the mutated hairpins (1L–5L and 1R–5R). The 23-nt target sequence complementary to the crRNA is highlighted in gray, and the mutated nucleotides are encircled. The thermodynamic stability (ΔG in kcal/mol) of both the WT target sequence and the artificial hairpins is indicated below the corresponding RNA structures.

against the predicted ΔG of the RNA target (Figures 2B and 2D). Cas13d targeting of the WT RdRp construct reduced the luciferase level by approximately 60% (from 100% to ∼40%), whereas targeting of the 1L and 1R variants reduced luciferase expression by less than 10% (from 100% to ∼90%). Similarly, targeting of the WT 5′ UTR-leader construct reduced the luciferase level approximately 60% (from 100% to ∼40%), whereas targeting of the 1L, 1R, and 2R variants reduced luciferase expression only approximately 30% (from 100% to ∼70%). Cas13d targeting of all other variants reduced the luciferase level to a level that was not significantly different from the level obtained for the corresponding WT construct. These results indicate that the very stable target RNA structures in the 1L and 1R RdRp variants and the 1L, 1R, and 2R 5′ UTR-leader variants demonstrate significant resistance to Cas13d cleavage. The target RNA structure is more stable in the 2R 5′ UTR-leader variant (ΔG −31.7 kcal/mol) than in the other 2R and 2L variants (ΔG −25.7 to

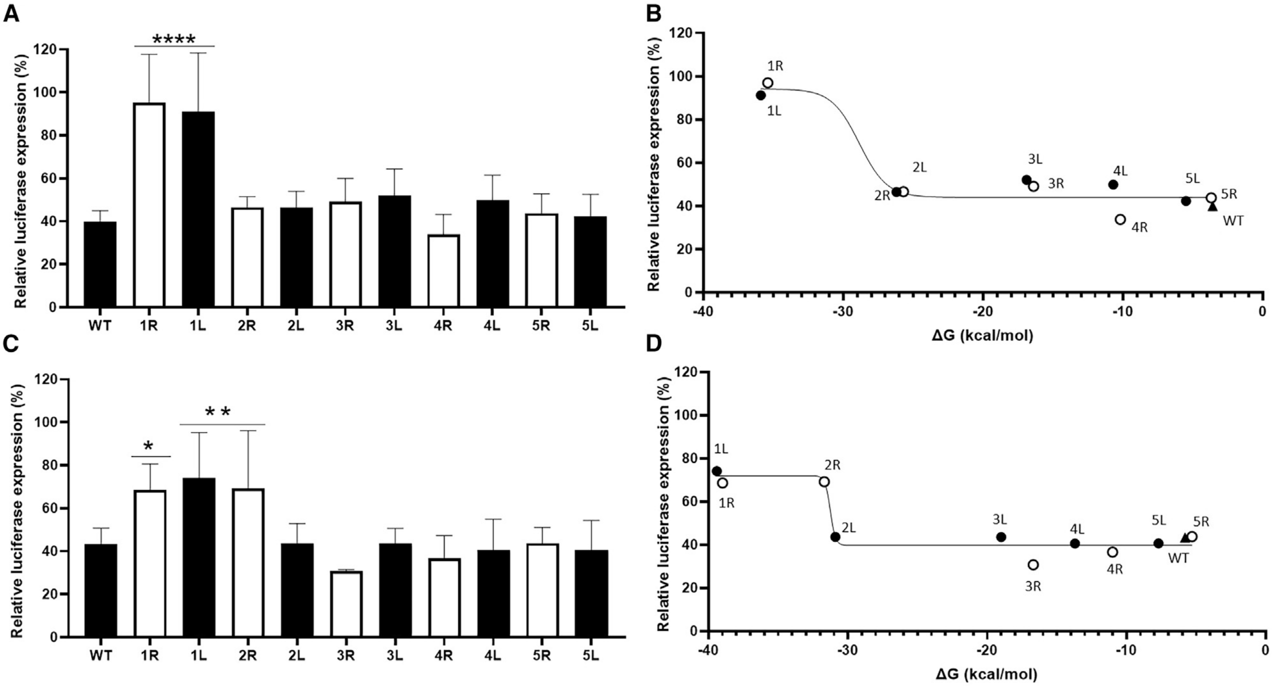

Figure 2. CRISPR-Cas13d targeting efficacy

The efficiency of CRISPR-Cas13d targeting of (A and B) RdRp- and (C and D) 5′ UTR-leader-encoding sequences was assessed in HEK293T cells. Cells were simultaneously transfected with the luciferase reporter constructs and Cas13d- and crRNA-expressing constructs (as shown in Figure 1), and the luciferase level was measured at 48 h post-transfection. (A and C) For every target variant, the luciferase activity measured with a non-targeting crRNA was set at 100%, and the relative luciferase level obtained with the targeting crRNA was calculated. The mean values (±SD) of three experiments performed in duplicate (*N* = 6) are presented. Statistical analysis using two-way ANOVA followed by Tukey's post hoc test was performed to identify statistically significant differences between the WT and mutant reporter constructs (**p* ≤ 0.05, ***p* ≤ 0.01, and *****p* ≤ 0.0001). (B and D) The mean relative luciferase expression levels as measured in (A) and (C) are plotted against the thermodynamic stability (ΔG in kcal/mol) of the WT (black triangles) and mutant target RNA structures (black and white circles representing 1L–5L and 1R–5R variants, respectively). Curves were generated with GraphPad Prism 9.1.0.

−30.9 kcal/mol). In these cell culture experiments, Cas13d targeting is thus inhibited only by very stable RNA structures (ΔG ≤ −31.7 kcal/mol).

Cas13d efficiently cleaves stable RNA hairpins

We next investigated the effect of target RNA structure on Cas13d cleavage in an *in vitro* cleavage assay. This assay relies on the Cas13d cleavage of the RNA target and the subsequent collateral ribonuclease activity of the Cas13d protein, which will lead to degradation of the cleaved RNA.[22] For this purpose, target RNA transcripts with the RdRp target sequence in a WT or mutated RNA hairpin structure (1R–4R; Figure 1B) were produced and incubated with a crRNA complementary to the RdRp sequence in the presence and absence of Cas13d protein. The samples were analyzed by denaturing agarose gel electrophoresis to visualize and quantify the target RNA (Figure 3A). When only the crRNA was added to the WT or mutant target RNAs, we observed both the target RNA (430 nt) and crRNA (54 nt) bands. When both the crRNA and Cas13d were added, the intensity of the target RNA bands was decreased for all variants, which indicates efficient Cas13d-mediated RNA cleavage. However, quantification of the target RNA bands revealed that Cas13d cleaved the 1R and 2R RNAs less efficiently than the WT, 3R, and 4R RNAs (Figure 3B), resulting in an approximately 2-fold increase in uncleaved target RNA signal. These *in vitro* experiments demonstrate that Cas13d cleavage is inhibited by the very stable local RNA structures present in the 1R and 2R variants (ΔG ≤ −25.7 kcal/mol) and not by the less stable structures present in the WT, 3R, and 4R variants.

RNAi-mediated RNA degradation is more sensitive to target RNA structure than Cas13d cleavage

We previously demonstrated the inhibitory effect of target RNA structure on RNAi efficiency.[37] For a more direct comparison of the sensitivity of Cas13d and RNAi for target RNA structure, we transfected cells with the WT and mutant SARS-CoV-2 luciferase reporter plasmids (Figure 1) and plasmids expressing either a short hairpin (sh)RNA targeting the RdRp or 5′ UTR-leader sequence or a non-targeting control shRNA that does not bind to any reporter construct or cellular sequence. The luciferase level was measured at 48 h post-transfection. For every target-reporter variant, the luciferase level measured with the control shRNA was set at 100%, and the relative luciferase level measured with the targeting shRNA was calculated. For all RdRp and 5′ UTR-leader constructs, the

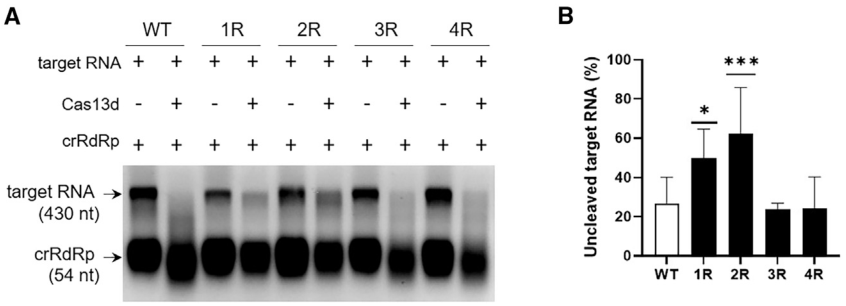

Figure 3. *In vitro* Cas13d cleavage assay

(A) *In vitro* cleavage of RdRp target RNAs using Cas13d protein and a crRNA targeting the RdRp sequence (crRdRp). WT and mutant (1R–4R) target RNAs were incubated with only the crRNA or with both the crRNA and Cas13d protein. The target RNAs and crRNAs were visualized by denaturing agarose gel electrophoresis. A representative gel image is shown, with the size of the RNAs shown on the left. (B) The target RNA bands observed without and with Cas13d addition were quantified with ImageJ software, and the percentage of uncleaved RNA observed upon Cas13d addition is shown (target RNA level with Cas13d/target RNA level without Cas13d × 100%). The bars represent the mean values (±SD) of 4 independent assays. Statistical analysis using two-way ANOVA followed by Tukey's post hoc test was performed to identify statistically significant differences between the WT and mutant target RNAs (*$p < 0.05$ and ***$p < 0.001$).

shRNA-mediated RNAi reduced the luciferase level, but the extent of this reduction varied significantly for the different constructs (Figure 4). Compared to the relatively low luciferase level measured for the WT RdRp construct (∼12%), we observed a significantly higher luciferase level for the 1R, 1L, 2R, and 2L variants (varying from 39% to 76%). Similarly, we observed a significantly higher luciferase level for the 1R, 1L, 2R, 2L, and 3L variants of the 5′ UTR-leader construct (varying from 36% to 82%) when compared to the low luciferase level measured for the WT construct (∼8%). These results indicate that the stabilized target RNA structure in these variants, with a ΔG varying from −39.4 to −19.0 kcal/mol, is more resistant to RNAi-induced cleavage than the WT target RNA structures (ΔG −3.6 and −5.8 kcal/mol). The luciferase level measured upon RNAi cleavage of the other variants did not differ (statistically) significantly from the level obtained with the WT constructs, indicating that the target RNA structures in these variants, with a ΔG varying from −16.9 to −3.7 kcal/mol, is similarly sensitive to RNAi cleavage as the WT target structure. These cell culture experiments thus demonstrate significant inhibition of RNAi by local RNA structures with a ΔG of at least −19.0 kcal/mol. As described above, inhibition of Cas13d activity in cell culture experiments with the same SARS-CoV-2 luciferase reporter constructs was only observed when this stability was at least −31.7 kcal/mol (Figure 2). These findings indicate that RNAi is more sensitive to local RNA structure than Cas13d.

DISCUSSION

The SARS-CoV-2 pandemic has sparked an extensive quest for antiviral strategies, including those derived from the CRISPR-Cas gene-editing toolkit. The CRISPR-Cas13 system that targets RNA has shown promise as a potential tool for combating SARS-CoV-2.[27,28] An important factor to consider when designing strategies that specifically target RNA is the impact of local RNA structure that may interfere with Cas13 action.[50] The possibility of suppression by local RNA structure becomes particularly important when targeting the RNA genome of SARS-CoV-2, as this is one of the most structured viral RNA genomes in nature.[44,45,51–55]

To investigate the influence of the local secondary RNA structure of the target sequence on Cas13d targeting in cell culture and *in vitro* experiments, we selected two potent anti-SARS-CoV-2 crRNAs from our previous study,[28] modified the sequence surrounding the actual target to create a perfect hairpin structure, and subsequently introduced specific mutations to gradually destabilize the hairpin (Figure 1). The findings demonstrate that Cas13d possesses the ability to effectively cleave RNA within fairly stable hairpin structures, and we only observed a significant decrease in Cas13d cleavage activity for RNA structures of exceptional stability (Figures 2 and 3). The position of the 23-nt target sequence (complementary to the crRNA) within the local RNA structure (L or R mutants in Figure 1) did not affect Cas13d cleavage. Although we observed, in general, a similar effect of the RNA stability on Cas13 activity in the cell and *in vitro* assay, some differences were observed. For example, we measured efficient cleavage of the 2R variant of the RdRp target (ΔG = −25.7 kcal/mol) in cells, whereas cleavage of this RNA was suppressed in the *in vitro* assay. Such differences are likely due to the distinct cleavage environment in the two assays. It is worth noting that perfect hairpins of 23 consecutive base pairs as we designed them with a ΔG < −25.7 kcal/mol, thus without any destabilizing bulges or internal loops, are relatively rare in nature.[56]

Several studies successfully employed RNAi as a strategy to hinder the replication and infection of SARS-CoV-2 and other viruses.[57–64] Suppressive effects of target RNA stability on RNAi efficiency have also been demonstrated.[37] To compare the sensitivity of Cas13d and RNAi for target RNA structures, we targeted the WT and stabilized SARS-CoV-2 target sequences also with RNAi in cell culture experiments, which revealed that RNAi was already inhibited by RNA structures with a ΔG of −19.0 kcal/mol (Figure 4), whereas Cas13d was only inhibited by more stable RNA structures (ΔG ≤ −31.7 kcal/mol; Figure 2). These results demonstrate that the Cas13d mechanism can tolerate stable RNA secondary structure better than the RNAi mechanism. It has been described that the compact binary Cas13:crRNA complex possesses an intrinsic capacity to unwind the RNA spacer, which may contribute to the higher tolerance of Cas13d in cleaving secondary structures.[65]

While both RNAi and CRISPR-Cas13d demonstrated efficacy as antivirals in preclinical studies, there are notable hurdles impeding their clinical application, with one of the primary challenges being the unintended silencing of off-target genes.[66–70] Both small interfering

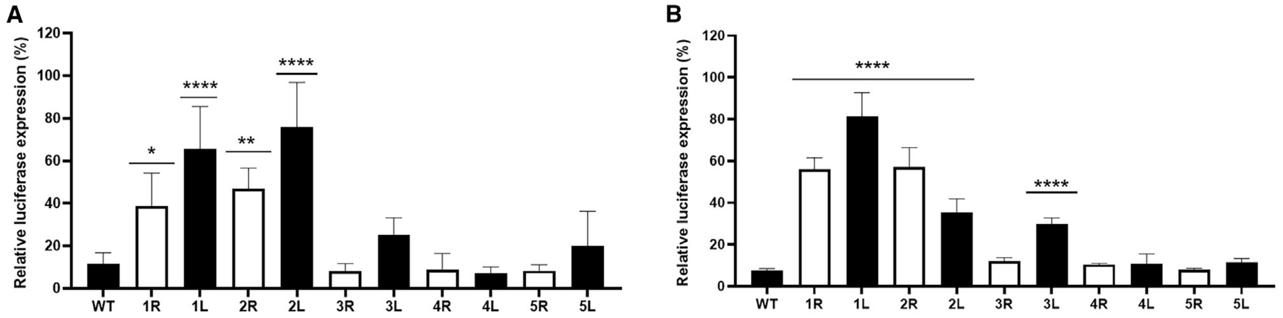

Figure 4. RNAi targeting efficacy

The efficiency of RNAi targeting of the WT and mutant RdRp (A) and 5′ UTR-leader (B) target RNA structures was assessed in HEK293T cells by co-transfection of the luciferase reporter constructs and shRNA expressing plasmids. Luciferase levels were measured 2 days after transfection. For every target variant, the luciferase level obtained with a non-targeting shRNA was set at 100%, and the relative luciferase level obtained with the targeting shRNA was calculated. The mean values (±SD) of three experiments performed in duplicate are presented ($N = 6$). Statistical analysis using two-way ANOVA followed by Tukey's post hoc test was performed to identify statistically significant differences between the WT and mutant reporter constructs (*$p \leq 0.05$, **$p \leq 0.01$, and ****$p \leq 0.0001$).

RNAs (siRNAs) and Cas13d/crRNAs can tolerate mismatches in the targeted complementary mRNAs.[69–71] However, Cas13d has demonstrated remarkable specificity, where mismatches in certain regions significantly impede its cleavage activity, allowing for the detection of SNPs.[72] The issue lies in the difficulty of accurately predicting such adverse events, necessitating extensive safety testing. Another significant difference between the two systems is related to their biological origin. The RNAi mechanism uses cellular components that enable microRNA (miRNA)-mediated regulation of cellular gene expression.[73] These components are inherently present in human cells. In contrast, CRISPR-Cas13d originates from prokaryotes, where it serves as a defense mechanism against invading bacteriophages. Therefore, not only the crRNA but also the relatively large and possibly immunogenic Cas13d protein should be supplied in applications in human cells. However, utilizing the endogenous RNAi system for therapeutic purposes also presents potential challenges, as the introduction of high levels of siRNAs may saturate the system and cause improper processing of (precursor) miRNAs,[74,75] which will affect cell physiology.

The results from our systematic analysis of the effect of stable target RNA structure on the Cas13d targeting efficiency underscore the potential of Cas13d as a tool for targeting viruses with a highly structured RNA genome, such as SARS-CoV-2 and hepatitis C virus. Moreover, these results may instruct other Cas13d applications. Future research should focus on the specific mechanism behind the low sensitivity of Cas13d for stable RNA structures.

MATERIALS AND METHODS

Plasmid constructs

The expression plasmid pLentiRNACRISPR_005-hU6-DR_BsmBI-EFS-RfxCas13d-NLS-2A-Puro-WPRE (Addgene, #138147) was designed to express the RfxCas13d endonuclease and crRNAs in mammalian cells. This construct was kindly donated by Neville Sanjana.[24] The EF1α core promoter controls the expression of the Cas13d nuclease, while the U6 polymerase III promoter was employed for transcription of the crRNAs. Oligonucleotides encoding SARS-CoV-2 targeting crRNAs and a non-targeting control crRNA (NC) were annealed and ligated into the Esp3I site of this vector. For the shRNA constructs, DNA oligonucleotides encoding the shRNA sequences were annealed and ligated into the BamHI and HindIII restriction sites of the pSilencer vector.[76] The crRNAs and shRNAs, including the non-targeting controls, are listed in Table S1. All constructs were verified by sequencing using the BigDye Terminator Cycle Sequencing kit (ABI) with a denaturation temperature of 98°C in the presence of 1 M betaine to disrupt structures in repeat sequence regions with high base-pairing potential such as crRNA and shRNA. For the construction of the luciferase reporter plasmids (Figure 1A), a 250-bp stuffer DNA fragment was inserted into the XbaI site downstream of the firefly luciferase coding region in the pGL3-control plasmid (Promega, GenBank: U47296.2) to introduce EcoRI and PstI sites.[42] Double-stranded DNA fragments encoding the differently structured target RNAs, prepared by combining the forward and reverse oligonucleotides listed in Table S2, were subsequently ligated into these EcoRI and PstI sites. All constructs were sequenced with the BigDye Terminator Cycle Sequencing kit (ABI).

The RNA secondary structure and ΔG of the 51-nt target RNA structures were predicted with the RNAfold webserver.[77]

Cell culture and luciferase assays

HEK293T cells were cultured in DMEM (Life Technologies, Invitrogen, Carlsbad, CA, USA) supplemented with 10% fetal calf serum, penicillin (100 U/mL), and streptomycin (100 mg/mL), 1% L-glutamine and MEM non-essential amino acids. The cells were cultured in a humidified chamber at 37°C and 5% CO_2. For luciferase assays, HEK293T cells were seeded 1 day prior to transfection in 24-well plates at a density of 1.4×10^5 cells per well in 0.5 mL media without antibiotics. Cells were transfected with 100 ng luciferase reporter plasmid, 1 ng of Renilla luciferase expression plasmid, and 300 ng of CRISPR-Cas13d/crRNA vector or 78 ng of shRNA vector using Lipofectamine 2000 reagent (Invitrogen) according to the

manufacturer's instructions. Firefly and Renilla luciferase levels were measured using the Dual-luciferase Reporter Assay System (Promega) at 2 days post-transfection, following the instructions provided by the manufacturer.[78] A non-targeting crRNA or shRNA was used as NC, and the luciferase level measured for this construct was set at 100%. We conducted three independent transfections, and each experiment was performed in duplicate. The ratio between firefly and Renilla luciferase activity was used to normalize for experimental variation. The luciferase data were then adjusted for inter-session variation using Factor Correction v.10.5.[79] The resulting six values were used to calculate the standard deviation. The data were analyzed using Prism software (GraphPad Prism 9.1.0). Two-way ANOVA followed by Tukey's post hoc test was used for all statistical analyses: $^{*}p \leq 0.05$, $^{**}p \leq 0.01$, $^{***}p \leq 0.001$, and $^{****}p \leq 0.0001$.

Production and purification of Cas13d protein

The Cas13d protein was obtained and purified according to previously described methods, with some adjustments.[80] *E. coli* Rosetta2 (DE3) competent cells were transformed with the pET-28b-RfxCas13d-His plasmid (Addgene, #141322) and grown in LB broth with 25 μg/mL kanamycin and 15 μg/mL chloramphenicol at 37°C until reaching an OD_{600} of 0.5. The cells were induced with 0.5 mM IPTG for 5 h, washed with 20 mM Tris-HCl (pH 7.6), pelleted, and stored at −80°C. For protein purification, the cell pellet was resuspended in lysis buffer containing 20 mM Tris-HCl (pH 7.5), 500 mM NaCl, 5 mM 2-mercaptoethanol, 10% v/v glycerol, 10 mM imidazole, and Pierce Protease Inhibitor (Thermo Scientific, A32963). The sample was subjected to five cycles of freezing in a 96% ethanol/dry-ice bath for 5 min and thawing in a 37°C water bath for 5 min. After sonication in a 10-s-on/20-s-off cycle for 5 min at 40% amplitude on ice, the lysate was centrifuged at 4,000 × *g* for 30 min at 4°C and filtered using a 0.22 μm cellulose acetate filter.

The Cas13d-His protein was purified using a HisTrap HP column (GE Healthcare) connected to a Peristaltic Pump P-1 (Cytiva). The column was washed with 5 column volume (CV) lysis buffer before the sample was loaded. The column was further washed with 4 CV washing buffer 1 (20 mM Tris-HCl [pH 7.5], 500 mM NaCl, 5 mM 2-mercaptoethanol, 10% v/v glycerol, 20 mM imidazole), followed by 8 CV washing buffer 2 (20 mM Tris-HCl [pH 7.5], 500 mM NaCl, 5 mM 2-mercaptoethanol, 10% v/v glycerol, 50 mM imidazole). The protein was dissociated from the column with a series of elution steps, firstly with 8 CV elution buffer 1 (20 mM Tris-HCl [pH 7.5], 500 mM NaCl, 5 mM 2-mercaptoethanol, 10% v/v glycerol, 75 mM imidazole) and subsequently with 4 CV elution buffer containing a gradually increasing concentration of imidazole (100, 125, 150, 175, and 200 mM).

The collected fractions were analyzed by SDS-PAGE, and those containing the recombinant protein were pooled. The samples were dialyzed at 4°C overnight against dialysis buffer (20 mM Tris-HCl [pH 7.5], 150 mM NaCl, 10% glycerol, and protease inhibitor) using Spectra/Por 3 Dialysis Tubing (3.5 kD MWCO, Repligen, 132720) and concentrated with a 50K ultra-filter (Merck, Millipore) at 4°C until the concentration reached 2 μg/μL. The protein concentration was measured using the NanoDrop One/Onec Microvolume UV-Vis Spectrophotometer (Thermo Scientific), and protein purity was analyzed by SDS-PAGE. Aliquots were stored at −80°C for future use.

***In vitro* transcription of target RNA**

Template DNAs were produced by PCR amplification of luciferase reporter plasmids using Phusion High-Fidelity DNA Polymerase and primers A2230-fwd (5′-C*TAATACGACTCACTATAG*GGCTATCA GGTGGCTCCCGCTGAATTGGAATCCATCTTG-3'; T7 RNA-polymerase promoter in italics) and A3674-rev (5′-GTGGTTTGT CCAAACTCATCAATG-3′), which yielded a 440-bp DNA product. The DNA products were purified using the QIAquick PCR Purification Kit, followed by *in vitro* transcription using the AmpliScribe T7-Flash In Vitro Transcription Kit (Epicenter, ASF3507). In brief, the DNA templates were mixed with dNTPs and T7 RNA polymerase following the instructions provided by the manufacturer, and the reactions were incubated for 2 h at 37°C. After *in vitro* transcription, the DNA template was digested with 1 μL Turbo DNase (2 U/μL) for 15 min at 37°C to remove any residual DNA, and the RNA was precipitated. For this, the samples (20 μL) were mixed with 80 μL nuclease-free water, 10 μL 3 M sodium acetate, and 300 μL ethanol, followed by incubation for 1 h at −80°C. The RNA was pelleted by centrifugation at 13,000 × *g* for 30 min at 4°C, and the pellet was washed twice with 75% ethanol followed by centrifugation at 13,000 × *g* for 10 min at 4°C before air drying the pellet for 10 min. The RNA was subsequently resuspended in 50 μL of RNase-free water.

***In vitro* cleavage assay**

The *in vitro* cleavage assay was performed following the protocol described by Konermann et al.[22] In brief, purified Cas13d protein (produced as described above) and crRNA (crRdRp, 5′- AACCC CUACCAACUGGUCGGGGUUUGAAACGCUAUGUAAGUUUA CAUCCUGAU-3′, RfxCas13d direct repeat [DR30] underlined; ordered from IDT) were mixed at a 1:1 M ratio in 20 μL RNA cleavage buffer (25 mM Tris [pH 7.5], 1 mM DTT, 6 mM $MgCl_2$) on ice and incubated at 37°C for 15 min. The target RNA was added at a 1:2 M ratio relative to Cas13d, and the mixture was incubated at 37°C for 45 min. Upon the addition of 1 μL stop solution (10 mg/mL proteinase K, 4 M urea, 80 mM EDTA, 20 mM Tris [pH 8.0]) and incubation at 37°C for 15 min, the volume was increased to 50 μL using RNase-free water, 1 μL of RNase-free glycogen (Thermo Scientific, R0551) was added as a carrier, and the RNA was purified using TRIzol extraction (Invitrogen, 15596018). For this, the RNA sample was mixed with 500 μL TRIzol, vortexed for 15 s, and incubated for 10 min at room temperature. Next, 100 μL of chloroform was added, and the mixture was vortexed for 15 s, followed by centrifugation at 7,500 × *g* for 10 min at 4°C, resulting in phase separation. The aqueous phase containing the RNA was mixed with 250 μL isopropanol. After 10 min on ice, the mixture was centrifuged for 10 min at 7,500 × *g* and 4°C. The resulting RNA pellet was washed with

500 μL ice-cold 75% ethanol and centrifuged for 5 min at 7,500 × *g* at 4°C. The RNA pellet was air dried for 10 min, followed by resuspension of the RNA in 10 μL of RNase-free water. Subsequently, 10 μL 2× RNA loading dye (Thermo Scientific, R0641) containing 10 μg/mL EtBr was added, and the RNA was denatured at 85°C for 8 min. After incubation on ice for 3 min, the samples were analyzed by denaturing formaldehyde-agarose gel electrophoresis at 100 V for 1 h. The RNA bands were visualized with UV light using a ProXima 2500 Gel Imager (Isogen Life Science). The target RNA signals were quantified with ImageJ software.[81]

Statistical analysis

All statistical analyses were performed using GraphPad Prism 9.1.0. The specifics of statistical analysis are noted in the figure legends.

DATA AND CODE AVAILABILITY

The data underlying this article are available in the article and in its online supplemental information.

SUPPLEMENTAL INFORMATION

Supplemental information can be found online at https://doi.org/10.1016/j.omtn.2024.102278.

ACKNOWLEDGMENTS

Y.L. is the recipient of a fellowship of the China Scholarship Council (CSC). This research is financially supported by the Netherlands Organization of Scientific Research (NWO) (Aspasia- 015.015.040).

AUTHOR CONTRIBUTIONS

E.H.-C. designed the project; M.H., Y.L., M.V., and P.Z.K. conducted the experiments; E.H.-C., M.H., Y.L., P.Z.K., B.B., and A.T.D. analyzed and studied the data; E.H.-C., M.H., Y.L., and A.T.D. wrote the original draft; and B.B. and P.Z.K. reviewed and edited the manuscript.

DECLARATION OF INTERESTS

The authors declare no competing interests.

REFERENCES

1. Ishino, Y., Shinagawa, H., Makino, K., Amemura, M., and Nakata, A. (1987). Nucleotide sequence of the iap gene, responsible for alkaline phosphatase isozyme conversion in Escherichia coli, and identification of the gene product. J. Bacteriol. *169*, 5429–5433.
2. Mojica, F.J.M., Juez, G., and Rodriguezvalera, F. (1993). Transcription at Different Salinities of Haloferax-Mediterranei Sequences Adjacent to Partially Modified Psti Sites. Mol. Microbiol. *9*, 613–621.
3. Mojica, F.J., Diez-Villasenor, C., Garcia-Martinez, J., and Soria, E. (2005). Intervening sequences of regularly spaced prokaryotic repeats derive from foreign genetic elements. J. Mol. Evol. *60*, 174–182.
4. Hsu, P.D., Lander, E.S., and Zhang, F. (2014). Development and applications of CRISPR-Cas9 for genome engineering. Cell *157*, 1262–1278.
5. Cong, L., Ran, F.A., Cox, D., Lin, S.L., Barretto, R., Habib, N., Hsu, P.D., Wu, X.B., Jiang, W.Y., Marraffini, L.A., and Zhang, F. (2013). Multiplex Genome Engineering Using CRISPR/Cas Systems. Science *339*, 819–823.
6. Zetsche, B., Gootenberg, J.S., Abudayyeh, O.O., Slaymaker, I.M., Makarova, K.S., Essletzbichler, P., Volz, S.E., Joung, J., van der Oost, J., Regev, A., et al. (2015). Cpf1 is a single RNA-guided endonuclease of a class 2 CRISPR-Cas system. Cell *163*, 759–771.
7. Teng, F., Cui, T., Feng, G., Guo, L., Xu, K., Gao, Q., Li, T., Li, J., Zhou, Q., and Li, W. (2018). Repurposing CRISPR-Cas12b for mammalian genome engineering. Cell Discov. *4*, 63.
8. Abudayyeh, O.O., Gootenberg, J.S., Konermann, S., Joung, J., Slaymaker, I.M., Cox, D.B.T., Shmakov, S., Makarova, K.S., Semenova, E., Minakhin, L., et al. (2016). C2c2 is a single-component programmable RNA-guided RNA-targeting CRISPR effector. Science *353*, aaf5573.
9. Shmakov, S., Abudayyeh, O.O., Makarova, K.S., Wolf, Y.I., Gootenberg, J.S., Semenova, E., Minakhin, L., Joung, J., Konermann, S., Severinov, K., et al. (2015). Discovery and Functional Characterization of Diverse Class 2 CRISPR-Cas Systems. Mol. Cell *60*, 385–397.
10. O'Connell, M.R. (2019). Molecular Mechanisms of RNA Targeting by Cas13-containing Type VI CRISPR-Cas Systems. J. Mol. Biol. *431*, 66–87.
11. East-Seletsky, A., O'Connell, M.R., Knight, S.C., Burstein, D., Cate, J.H., Tjian, R., and Doudna, J.A. (2016). Two distinct RNase activities of CRISPR-C2c2 enable guide-RNA processing and RNA detection. Nature *538*, 270–273.
12. Zhang, B., Ye, Y.M., Ye, W.W., Perculija, V., Jiang, H., Chen, Y.Y., Li, Y., Chen, J., Lin, J.Y., Wang, S.Q., et al. (2019). Two HEPN domains dictate CRISPR RNA maturation and target cleavage in Cas13d. Nat. Commun. *10*, 2544.
13. Kordyś, M., Sen, R., and Warkocki, Z. (2022). Applications of the versatile CRISPR-Cas13 RNA targeting system. Wiley Interdiscip. Rev. RNA *13*, e1694.
14. Knott, G.J., East-Seletsky, A., Cofsky, J.C., Holton, J.M., Charles, E., O'Connell, M.R., and Doudna, J.A. (2017). Guide-bound structures of an RNA-targeting A-cleaving CRISPR-Cas13a enzyme. Nat. Struct. Mol. Biol. *24*, 825–833.
15. Cox, D.B.T., Gootenberg, J.S., Abudayyeh, O.O., Franklin, B., Kellner, M.J., Joung, J., and Zhang, F. (2017). RNA editing with CRISPR-Cas13. Science *358*, 1019–1027.
16. Meeske, A.J., Nakandakari-Higa, S., and Marraffini, L.A. (2019). Cas13-induced cellular dormancy prevents the rise of CRISPR-resistant bacteriophage. Nature *570*, 241–245.
17. Gootenberg, J.S., Abudayyeh, O.O., Lee, J.W., Essletzbichler, P., Dy, A.J., Joung, J., Verdine, V., Donghia, N., Daringer, N.M., Freije, C.A., et al. (2017). Nucleic acid detection with CRISPR-Cas13a/C2c2. Science *356*, 438–442.
18. He, B., Peng, W., Huang, J., Zhang, H., Zhou, Y., Yang, X., Liu, J., Li, Z., Xu, C., Xue, M., et al. (2020). Modulation of metabolic functions through Cas13d-mediated gene knockdown in liver. Protein Cell *11*, 518–524.
19. Zhou, C., Hu, X., Tang, C., Liu, W., Wang, S., Zhou, Y., Zhao, Q., Bo, Q., Shi, L., Sun, X., et al. (2020). CasRx-mediated RNA targeting prevents choroidal neovascularization in a mouse model of age-related macular degeneration. Natl. Sci. Rev. *7*, 835–837.
20. Powell, J.E., Lim, C.K.W., Krishnan, R., McCallister, T.X., Saporito-Magrina, C., Zeballos, M.A., McPheron, G.D., and Gaj, T. (2022). Targeted gene silencing in the nervous system with CRISPR-Cas13. Sci. Adv. *8*, eabk2485.
21. Li, Y., Xu, J., Guo, X., Li, Z., Cao, L., Liu, S., Guo, Y., Wang, G., Luo, Y., Zhang, Z., and Wei, X. (2023). The collateral activity of RfxCas13d can induce lethality in a RfxCas13d knock-in mouse model. Genome Biol. *24*, 20.
22. Konermann, S., Lotfy, P., Brideau, N.J., Oki, J., Shokhirev, M.N., and Hsu, P.D. (2018). Transcriptome Engineering with RNA-Targeting Type VI-D CRISPR Effectors. Cell *173*, 665–676.e14.
23. Smargon, A.A., Cox, D.B.T., Pyzocha, N.K., Zheng, K., Slaymaker, I.M., Gootenberg, J.S., Abudayyeh, O.A., Essletzbichler, P., Shmakov, S., Makarova, K.S., et al. (2017). Cas13b Is a Type VI-B CRISPR-Associated RNA-Guided RNase Differentially Regulated by Accessory Proteins Csx27 and Csx28. Mol. Cell *65*, 618–630.e7.
24. Wessels, H.H., Méndez-Mancilla, A., Guo, X., Legut, M., Daniloski, Z., and Sanjana, N.E. (2020). Massively parallel Cas13 screens reveal principles for guide RNA design. Nat. Biotechnol. *38*, 722–727.
25. Lotfi, M., and Rezaei, N. (2020). CRISPR/Cas13: A potential therapeutic option of COVID-19. Biomed. Pharmacother. *131*, 110738.
26. Bayoumi, M., and Munir, M. (2021). Potential Use of CRISPR/Cas13 Machinery in Understanding Virus-Host Interaction. Front. Microbiol. *12*, 743580.

27. Abbott, T.R., Dhamdhere, G., Liu, Y., Lin, X., Goudy, L., Zeng, L., Chemparathy, A., Chmura, S., Heaton, N.S., Debs, R., et al. (2020). Development of CRISPR as an Antiviral Strategy to Combat SARS-CoV-2 and Influenza. Cell *181*, 865–876.e12.

28. Hussein, M., Andrade Dos Ramos, Z., Vink, M.A., Kroon, P., Yu, Z., Enjuanes, L., Zuniga, S., Berkhout, B., and Herrera-Carrillo, E. (2023). Efficient CRISPR-Cas13d-Based Antiviral Strategy to Combat SARS-CoV-2. Viruses *15*, 686.

29. Blanchard, E.L., Vanover, D., Bawage, S.S., Tiwari, P.M., Rotolo, L., Beyersdorf, J., Peck, H.E., Bruno, N.C., Hincapie, R., Michel, F., and Murray, J. (2021). Treatment of influenza and SARS-CoV-2 infections via mRNA-encoded Cas13a in rodents. Nat. Biotechnol. *39*, 717–726.

30. Joshi, G., Borah, P., Thakur, S., Sharma, P., Mayank, and Poduri, R. (2021). Exploring the COVID-19 vaccine candidates against SARS-CoV-2 and its variants: where do we stand and where do we go? Hum. Vaccines Immunother. *17*, 4714–4740.

31. Li, M., Wang, H., Tian, L., Pang, Z., Yang, Q., Huang, T., Fan, J., Song, L., Tong, Y., and Fan, H. (2022). COVID-19 vaccine development: milestones, lessons and prospects. Signal Transduct. Targeted Ther. *7*, 146.

32. van Gils, M.J., Lavell, A., van der Straten, K., Appelman, B., Bontjer, I., Poniman, M., Burger, J.A., Oomen, M., Bouhuijs, J.H., van Vught, L.A., et al. (2022). Antibody responses against SARS-CoV-2 variants induced by four different SARS-CoV-2 vaccines in health care workers in the Netherlands: A prospective cohort study. PLoS Med. *19*, e1003991.

33. Abudayyeh, O.O., Gootenberg, J.S., Essletzbichler, P., Han, S., Joung, J., Belanto, J.J., Verdine, V., Cox, D.B.T., Kellner, M.J., Regev, A., et al. (2017). RNA targeting with CRISPR-Cas13. Nature *550*, 280–284.

34. Hussein, M., Andrade Dos Ramos, Z., Berkhout, B., and Herrera-Carrillo, E. (2022). In Silico Prediction and Selection of Target Sequences in the SARS-CoV-2 RNA Genome for an Antiviral Attack. Viruses *14*, 385.

35. ter Brake, O., von Eije, K.J., and Berkhout, B. (2008). Probing the sequence space available for HIV-1 evolution. AIDS *22*, 1875–1877.

36. von Eije, K.J., ter Brake, O., and Berkhout, B. (2008). Human immunodeficiency virus type 1 escape is restricted when conserved genome sequences are targeted by RNA interference. J. Virol. *82*, 2895–2903.

37. Westerhout, E.M., and Berkhout, B. (2007). A systematic analysis of the effect of target RNA structure on RNA interference. Nucleic Acids Res. *35*, 4322–4330.

38. Ameres, S.L., Martinez, J., and Schroeder, R. (2007). Molecular basis for target RNA recognition and cleavage by human RISC. Cell *130*, 101–112.

39. Shao, Y., Chan, C.Y., Maliyekkel, A., Lawrence, C.E., Roninson, I.B., and Ding, Y. (2007). Effect of target secondary structure on RNAi efficiency. RNA *13*, 1631–1640.

40. Tafer, H., Ameres, S.L., Obernosterer, G., Gebeshuber, C.A., Schroeder, R., Martinez, J., and Hofacker, I.L. (2008). The impact of target site accessibility on the design of effective siRNAs. Nat. Biotechnol. *26*, 578–583.

41. Vickers, T.A., Wyatt, J.R., and Freier, S.M. (2000). Effects of RNA secondary structure on cellular antisense activity. Nucleic Acids Res. *28*, 1340–1347.

42. Westerhout, E.M., Ooms, M., Vink, M., Das, A.T., and Berkhout, B. (2005). HIV-1 can escape from RNA interference by evolving an alternative structure in its RNA genome. Nucleic Acids Res. *33*, 796–804.

43. Bandaru, S., Tsuji, M.H., Shimizu, Y., Usami, K., Lee, S., Takei, N.K., Yoshitome, K., Nishimura, Y., Otsuki, T., and Ito, T. (2020). Structure-based design of gRNA for Cas13. Sci. Rep. *10*, 11610.

44. Tavares, R.D.C.A., Mahadeshwar, G., Wan, H., Huston, N.C., and Pyle, A.M. (2020). The global and local distribution of RNA structure throughout the SARS-CoV-2 genome. J. Virol. *95*, 10–1128.

45. Manfredonia, I., Nithin, C., Ponce-Salvatierra, A., Ghosh, P., Wirecki, T.K., Marinus, T., Ogando, N.S., Snijder, E.J., van Hemert, M.J., Bujnicki, J.M., and Incarnato, D. (2020). Genome-wide mapping of SARS-CoV-2 RNA structures identifies therapeutically-relevant elements. Nucleic Acids Res. *48*, 12436–12452.

46. Andrews, R.J., Peterson, J.M., Haniff, H.S., Chen, J., Williams, C., Grefe, M., Disney, M.D., and Moss, W.N. (2020). An in silico map of the SARS-CoV-2 RNA Structurome. Preprint at bioRxiv *48*, 12436. https://academic.oup.com/nargab/article/3/2/lqab043/6281450.

47. Andrews, R.J., O'Leary, C.A., Tompkins, V.S., Peterson, J.M., Haniff, H.S., Williams, C., Disney, M.D., and Moss, W.N. (2021). A map of the SARS-CoV-2 RNA structurome. NAR Genom. Bioinform. *3*, lqab043.

48. Bassett, M., Salemi, M., and Rife Magalis, B. (2022). Lessons Learned and Yet-to-Be Learned on the Importance of RNA Structure in SARS-CoV-2 Replication. Microbiol. Mol. Biol. Rev. *86*, e0005721.

49. Ahmed, F., Sharma, M., Al-Ghamdi, A.A., Al-Yami, S.M., Al-Salami, A.M., Refai, M.Y., Warsi, M.K., Howladar, S.M., and Baeshen, M.N. (2020). A Comprehensive Analysis of cis-Acting RNA Elements in the SARS-CoV-2 Genome by a Bioinformatics Approach. Front. Genet. *11*, 572702.

50. Ganser, L.R., Kelly, M.L., Herschlag, D., and Al-Hashimi, H.M. (2019). The roles of structural dynamics in the cellular functions of RNAs. Nat. Rev. Mol. Cell Biol. *20*, 474–489.

51. Yang, D., and Leibowitz, J.L. (2015). The structure and functions of coronavirus genomic 3' and 5' ends. Virus Res. *206*, 120–133.

52. Madhugiri, R., Fricke, M., Marz, M., and Ziebuhr, J. (2016). Coronavirus cis-Acting RNA Elements. Adv. Virus Res. *96*, 127–163.

53. Goebel, S.J., Hsue, B., Dombrowski, T.F., and Masters, P.S. (2004). Characterization of the RNA components of a putative molecular switch in the 3' untranslated region of the murine coronavirus genome. J. Virol. *78*, 669–682.

54. Plant, E.P., and Dinman, J.D. (2008). The role of programmed-1 ribosomal frameshifting in coronavirus propagation. Front. Biosci. *13*, 4873–4881.

55. Plant, E.P., Sims, A.C., Baric, R.S., Dinman, J.D., and Taylor, D.R. (2013). Altering SARS coronavirus frameshift efficiency affects genomic and subgenomic RNA production. Viruses *5*, 279–294.

56. Varani, G. (1995). Exceptionally stable nucleic acid hairpins. Annu. Rev. Biophys. Biomol. Struct. *24*, 379–404.

57. Ambike, S., Cheng, C.C., Feuerherd, M., Velkov, S., Baldassi, D., Afridi, S.Q., Porras-Gonzalez, D., Wei, X., Hagen, P., Kneidinger, N., et al. (2022). Targeting genomic SARS-CoV-2 RNA with siRNAs allows efficient inhibition of viral replication and spread. Nucleic Acids Res. *50*, 333–349.

58. Lee, Y.R., Tsai, H.P., Yeh, C.S., Fang, C.Y., Chan, M.W., Wu, T.Y., and Shen, C.H. (2022). RNA Interference Approach Is a Good Strategy against SARS-CoV-2. Viruses *15*, 100.

59. Idris, A., Davis, A., Supramaniam, A., Acharya, D., Kelly, G., Tayyar, Y., West, N., Zhang, P., McMillan, C.L.D., Soemardy, C., et al. (2021). A SARS-CoV-2 targeted siRNA-nanoparticle therapy for COVID-19. Mol. Ther. *29*, 2219–2226.

60. Becker, J., Stanifer, M.L., Leist, S.R., Stolp, B., Maiakovska, O., West, A., Wiedtke, E., Borner, K., Ghanem, A., Ambiel, I., et al. (2022). Ex vivo and *in vivo* suppression of SARS-CoV-2 with combinatorial AAV/RNAi expression vectors. Mol. Ther. *30*, 2005–2023.

61. Chang, Y.C., Yang, C.F., Chen, Y.F., Yang, C.C., Chou, Y.L., Chou, H.W., Chang, T.Y., Chao, T.L., Hsu, S.C., Ieong, S.M., et al. (2022). A siRNA targets and inhibits a broad range of SARS-CoV-2 infections including Delta variant. EMBO Mol. Med. *14*, e15298.

62. Baldassi, D., Ambike, S., Feuerherd, M., Cheng, C.C., Peeler, D.J., Feldmann, D.P., Porras-Gonzalez, D.L., Wei, X., Keller, L.A., Kneidinger, N., et al. (2022). Inhibition of SARS-CoV-2 replication in the lung with siRNA/VIPER polyplexes. J. Contr. Release *345*, 661–674.

63. Kelleher, A.D., Cortez-Jugo, C., Cavalieri, F., Qu, Y., Glanville, A.R., Caruso, F., Symonds, G., and Ahlenstiel, C.L. (2020). RNAi therapeutics: an antiviral strategy for human infections. Curr. Opin. Pharmacol. *54*, 121–129.

64. Lundstrom, K. (2020). Viral Vectors Applied for RNAi-Based Antiviral Therapy. Viruses *12*, 924.

65. Liu, L., and Pei, D.S. (2022). Insights Gained from RNA Editing Targeted by the CRISPR-Cas13 Family. Int. J. Mol. Sci. *23*, 11400.

66. Jackson, A.L., Bartz, S.R., Schelter, J., Kobayashi, S.V., Burchard, J., Mao, M., Li, B., Cavet, G., and Linsley, P.S. (2003). Expression profiling reveals off-target gene regulation by RNAi. Nat. Biotechnol. *21*, 635–637.

67. Sudbery, I., Enright, A.J., Fraser, A.G., and Dunham, I. (2010). Systematic analysis of off-target effects in an RNAi screen reveals microRNAs affecting sensitivity to TRAIL-induced apoptosis. BMC Genom. *11*, 175.

68. Chen, J., Peng, Y., Zhang, H., Wang, K., Zhao, C., Zhu, G., Reddy Palli, S., and Han, Z. (2021). Off-target effects of RNAi correlate with the mismatch rate between dsRNA and non-target mRNA. RNA Biol. *18*, 1747–1759.

69. Ui-Tei, K. (2016). Is the Efficiency of RNA Silencing Evolutionarily Regulated? Int. J. Mol. Sci. *17*, 719.

70. Wessels, H.H., Stirn, A., Mendez-Mancilla, A., Kim, E.J., Hart, S.K., Knowles, D.A., and Sanjana, N.E. (2024). Prediction of on-target and off-target activity of CRISPR-Cas13d guide RNAs using deep learning. Nat. Biotechnol. *42*, 628–637.

71. Ahmed, F., and Raghava, G.P. (2011). Designing of highly effective complementary and mismatch siRNAs for silencing a gene. PLoS One *6*, e23443.

72. Kuo, H.C., Prupes, J., Chou, C.W., and Finkelstein, I.J. (2023). Massively Parallel Profiling of RNA-targeting CRISPR-Cas13d. Preprint at bioRxiv *48*, 12436. https://www.nature.com/articles/s41467-024-44738-w.

73. Kim, D., and Rossi, J. (2008). RNAi mechanisms and applications. Biotechniques *44*, 613–616.

74. Raja, M.A.G., Katas, H., and Amjad, M.W. (2019). Design, mechanism, delivery and therapeutics of canonical and Dicer-substrate siRNA. Asian J. Pharm. Sci. *14*, 497–510.

75. Alshaer, W., Zureigat, H., Al Karaki, A., Al-Kadash, A., Gharaibeh, L., Hatmal, M.M., Aljabali, A.A.A., and Awidi, A. (2021). siRNA: Mechanism of action, challenges, and therapeutic approaches. Eur. J. Pharmacol. *905*, 174178.

76. Brummelkamp, T.R., Bernards, R., and Agami, R. (2002). A system for stable expression of short interfering RNAs in mammalian cells. Science *296*, 550–553.

77. Vienna, Uo RNAfold web server http://rna.tbi.univie.ac.at/cgi-bin/RNAWebSuite/RNAfold.cgi.

78. Promega Dual-Luciferase® Reporter Assay System https://nld.promega.com/products/luciferase-assays/reporter-assays/dual_luciferase-reporter-assay-system/?catNum=E1910.

79. Ruijter, J.M., Thygesen, H.H., Schoneveld, O.J., Das, A.T., Berkhout, B., and Lamers, W.H. (2006). Factor correction as a tool to eliminate between-session variation in replicate experiments: application to molecular biology and retrovirology. Retrovirology *3*, 2.

80. Kushawah, G., Hernandez-Huertas, L., Del Prado, J.A.N., Martinez-Morales, J.R., DeVore, M.L., Hassan, H., Moreno-Sanchez, I., Tomas-Gallardo, L., Diaz-Moscoso, A., Monges, D.E., et al. (2020). CRISPR-Cas13d Induces Efficient mRNA Knockdown in Animal Embryos. Dev. Cell *54*, 805–817.

81. Schneider, C.A., Rasband, W.S., and Eliceiri, K.W. (2012). NIH Image to ImageJ: 25 years of image analysis. Nat. Methods *9*, 671–675.

OMTN, Volume *35*

Supplemental information

Evaluation of the effect of RNA secondary structure on Cas13d-mediated target RNA cleavage

Mouraya Hussein, Ye Liu, Monique Vink, Pascal Z. Kroon, Atze T. Das, Ben Berkhout, and Elena Herrera-Carrillo

Table S1. DNA oligonucleotides

Oligonucleotide name	Oligonucleotide sequence
FWD 5'UTR-Leader-crRNA	AAACGAGAACAGATCTACAAGAGATCG
REV 5'UTR-Leader-crRNA	AAAACGATCTCTTGTAGATCTGTTCTC
FWD RdRp-crRNA	AAACGCTATGTAAGTTTACATCCTGAT
REV RdRp-crRNA	AAAAATCAGGATGTAAACTTACATAGC
FWD-NC-crRNA	AAACGAGGAGGTACTGCTGCGATGGGA
REV-NC-crRNA	AAAATCCCATCGCAGCAGTACCTCCTC
FWD 5'UTR-Leader-shRNA	AGCTTTTCCAAAAAACGATCTCTTGTAGATCTGTTCTCTCTTGAAGAACAGATCTACAAGAGATCGGGG
REV 5'UTR-Leader-shRNA	GATCCCCCGATCTCTTGTAGATCTGTTCTTCAAGAGAGAACAGATCTACAAGAGATCGTTTTTTGGAAA
FWD RdRp-shRNA	AGCTTTTCCAAAAAAATCAGGATGTAAACTTACATATCTCTTGAATATGTAAGTTTACATCCTGATGGG
REV RdRp-shRNA	GATCCCCATCAGGATGTAAACTTACATATTCAAGAGATATGTAAGTTTACATCCTGATTTTTTTGGAAA
FWD-NC-shRNA	AGCTTTTCCAAAAAATCCCATCGCAGCAGTACCTCCTCTCTTGAAGGAGGTACTGCTGCGATGGGAGGG
REV-NC-shRNA	GATCCCCTCCCATCGCAGCAGTACCTCCTTCAAGAGAGGAGGTACTGCTGCGATGGGATTTTTTGGAAA

Table S2. Target oligonucleotide sequences

Oligonucleotide name	Oligonucleotide sequence
RdRp.WT.FWD	AATTCAGGTGTTGTACATAATCAGGATGTAAACTTACATAGCTCTAGACTTAGTTTCTGCA
RdRp.WT.Rev	GAAACTAAGTCTAGAGCTATGTAAGTTTACATCCTGATTATGTACAACACCTG
RdRp.MUT1R.FWD	AATTCGCTATGTAAGTTTACATCCTGATaagaaATCAGGATGTAAACTTACATAGCCTGCA
RdRp.MUT1R.Rev	GGCTATGTAAGTTTACATCCTGATttcttATCAGGATGTAAACTTACATAGCG
RdRp.MUT2R.FWD	AATTCGTTATGTAAGTTTATATTTTGATaagaaATCAGGATGTAAACTTACATAGCCTGCA
RdRp.MUT2R.Rev	GGCTATGTAAGTTTACATCCTGATttcttATCAAAATATAAACTTACATAACG
RdRp.Mut3R.FWD	AATTCGCTATGTAAATTTAAATCATGATaagaaATCAGGATGTAAACTTACATAGCCTGCA
RdRp.Mut3R.Rev	GGCTATGTAAGTTTACATCCTGATttcttATCATGATTTAAATTTACATAGCG
RdRp.Mut4R.FWD	AATTCGCTCAGTAAATTTAAATCATGATaagaaATCAGGATGTAAACTTACATAGCCTGCA
RdRp.Mut4R.Rev	GGCTATGTAAGTTTACATCCTGATttcttATCATGATTTAAATTTACTGAGCG
RdRp.Mut5R.FWD	AATTCGCAATGCAAATCTAAATCATGAAaagaaATCAGGATGTAAACTTACATAGCCTGCA
RdRp.Mut5R.Rev	GGCTATGTAAGTTTACATCCTGATttcttTTCATGATTTAGATTTGCATTGCG
RdRp.Mut1L.FWD	AATTCATCAGGATGTAAACTTACATAGCaagaaGCTATGTAAGTTTACATCCTGATCTGCA
RdRp.Mut1L.Rev	GATCAGGATGTAAACTTACATAGCttcttGCTATGTAAGTTTACATCCTGATG
RdRp.Mut2L.FWD	AATTCATCAGGATGTAAACTTACATAGCaagaaGTTATGTAAGTTTATATTTTGATCTGCA
RdRp.Mut2L.Rev	GATCAAAATATAAACTTACATAACttcttGCTATGTAAGTTTACATCCTGATG
RdRp.Mut3L.FWD	AATTCATCAGGATGTAAACTTACATAGCaagaaGCTATGTAAATTTAAATCATGATCTGCA
RdRp.Mut3L.Rev	GATCATGATTTAAATTTACATAGCttcttGCTATGTAAGTTTACATCCTGATG
RdRp.Mut4L.FWD	AATTCATCAGGATGTAAACTTACATAGCaagaaGCTCAGTAAATTTAAATCATGATCTGCA
RdRp.Mut4L.Rev	GATCATGATTTAAATTTACTGAGCttcttGCTATGTAAGTTTACATCCTGATG
RdRp.Mut5L.FWD	AATTCATCAGGATGTAAACTTACATAGCaagaaGCAATGCAAATCTAAATCATGAACTGCA
RdRp.Mut5L.Rev	GTTCATGATTTAGATTTGCATTGCttcttGCTATGTAAGTTTACATCCTGATG
5'UTR-Leader.WT.FWD	AATTCAACCAACCAACTTTCGATCTCTTGTAGATCTGTTCTCTAAACGAACTTTAACTGCA
5'UTR-Leader.WT.Rev	GTTAAAGTTCGTTTAGAGAACAGATCTACAAGAGATCGAAAGTTGGTTGGTTG
5'UTR-Leader.Mut1R.FWD	AATTCGAGAACAGATCTACAAGAGATCGaagaaCGATCTCTTGTAGATCTGTTCTCCTGCA
5'UTR-Leader.Mut1R.Rev	GGAGAACAGATCTACAAGAGATCGttcttCGATCTCTTGTAGATCTGTTCTCG
5'UTR-Leader.Mut2R.FWD	AATTCGAGAAtAGATtTAtAAGAGATtGcaccaCGATCTCTTGTAGATCTGTTCTCCTGCA
5'UTR-Leader.Mut2R.Rev	GGAGAACAGATCTACAAGAGATCGtggtgCaATCTCTTaTAaATCTaTTCTCG
5'UTR-Leader.Mut3R.FWD	AATTCGAGAAaAGATaTAgAAGAGATaGcaccaCGATCTCTTGTAGATCTGTTCTCCTGCA
5'UTR-Leader.Mut3R.Rev	GGAGAACAGATCTACAAGAGATCGtggtgCtATCTCTTcTAtATCTtTTCTCG
5'UTR-Leader.Mut4R.FWD	AATTCGAGAtaAGATaTAgAAGAaATaGcaccaCGATCTCTTGTAGATCTGTTCTCCTGCA
5'UTR-Leader.Mut4R.Rev	GGAGAACAGATCTACAAGAGATCGtggtgCtATtTCTTcTAtATCTtaTCTCG
5'UTR-Leader.Mut5R.FWD	AATTCGAtAtaAGATaTAgAAGAaATaGcaccaCGATCTCTTGTAGATCTGTTCTCCTGCA
5'UTR-Leader.Mut5R.Rev	GGAGAACAGATCTACAAGAGATCGtggtgCtATtTCTTcTAtATCTtaTaTCG
5'UTR-Leader.Mut1L.FWD	AATTCCGATCTCTTGTAGATCTGTTCTCaagaaGAGAACAGATCTACAAGAGATCGCTGCA
5'UTR-Leader.Mut1L.Rev	GCGATCTCTTGTAGATCTGTTCTCttcttGAGAACAGATCTACAAGAGATCGG
5'UTR-Leader.Mut2L.FWD	AATTCCGATCTCTTGTAGATCTGTTCTCaagaaGAGAAtAGATtTAtAAGAGATtGCTGCA
5'UTR-Leader.Mut2L.Rev	GCaATCTCTTaTAaATCTaTTCTCttcttGAGAACAGATCTACAAGAGATCGG
5'UTR-Leader.Mut3L.FWD	AATTCCGATCTCTTGTAGATCTGTTCTCaagaaGAGAAaAGATaTAgAAGAGATaGCTGCA
5'UTR-Leader.Mut3L.Rev	GCtATCTCTTcTAtATCTtTTCTCttcttGAGAACAGATCTACAAGAGATCGG
5'UTR-Leader.Mut4L.FWD	AATTCCGATCTCTTGTAGATCTGTTCTCaagaaGAGAtaAGATaTAgAAGAaATaGCTGCA
5'UTR-Leader.Mut4L.Rev	GCtATtTCTTcTAtATCTtaTCTCttcttGAGAACAGATCTACAAGAGATCGG
5'UTR-Leader.Mut5L.FWD	AATTCCGATCTCTTGTAGATCTGTTCTCcaccaGAtAtaAGATaTAgAAGAaATaGCTGCA
5'UTR-Leader.Mut5L.Rev	GCtATtTCTTcTAtATCTtaTaTCtggtgGAGAACAGATCTACAAGAGATCGG
